# Food Environments and Food Security Among Mothers and Children in Northeast Brazil: The Role of Social and Housing Conditions

**DOI:** 10.3390/ijerph23070936

**Published:** 2026-07-22

**Authors:** Verônyky Gomes da Silva, Nathalia Barbosa de Aquino, Maria Suzane Barbosa, Larissa de Lima Soares, Risia Cristina Egito de Menezes, Juliana Souza Oliveira

**Affiliations:** 1 Graduate Program in Nutrition, Health Sciences Center, Federal University of Pernambuco, Recife 50670-901, PE, Brazil; nathalia.aquino@ufpe.br (N.B.d.A.); maria.suzaneb@ufpe.br (M.S.B.); juliana.souzao@ufpe.br (J.S.O.); 2 Graduate Program in Nutrition, Faculty of Nutrition, Federal University of Alagoas, Maceió 57072-970, AL, Brazil; larissa.soares@fanut.ufal.br (L.d.L.S.); risia.menezes@fanut.ufal.br (R.C.E.d.M.); 3 Nutrition Program, Vitória Academic Center, Federal University of Pernambuco, Vitória de Santo Antão 55608-680, PE, Brazil

**Keywords:** social determinants, food environment, food security, nutritional status, structural equation modeling

## Abstract

**Highlights:**

**Public health relevance—How does this work relate to a public health issue?**

- Social and housing conditions were associated with household food security among mothers and children in a socially vulnerable setting in Northeast Brazil.
- Perceived food environments showed limited associations within the proposed model and did not mediate the relationship between social and housing conditions and household food security.

**Public health significance—Why is this work of significance to public health?**

- More favorable social and housing conditions were associated with healthier domestic food environments and lower food insecurity among vulnerable populations.
- Associations between food security, maternal BMI, and child BMI highlight the nutritional consequences of food insecurity across generations.

**Public health implications—What are the key implications or messages for practitioners, policy makers and/or researchers in public health?**

- Policies addressing social and housing inequalities should be integrated into food security strategies, particularly in socially vulnerable settings, as interventions focused exclusively on community food environments may have limited impact if these broader inequalities are not addressed simultaneously.
- Future research should investigate additional pathways linking social and housing conditions to food security and nutritional outcomes, including objective measures of food environments and longitudinal designs.

**Abstract:**

**Background:** Perceived characteristics of the food environment may influence individuals’ dietary practices and health outcomes. This study analyzed associations between social and housing conditions, food environment perception, and food security, and their associations with maternal and child nutritional status. **Methods**: A cross-sectional study conducted between 2022 and 2023 with 314 participants in Vitória de Santo Antão, Pernambuco, Brazil. Socioeconomic variables, housing conditions, perceptions of domestic and community food environments, food security, and anthropometric indicators were assessed using generalized structural equation modeling (GSEM, *p* < 0.05). **Results:** Better social and housing conditions were associated with a more favorable perception of the domestic food environment (β = 0.79; 95% CI: 0.23–1.34) and lower food insecurity severity (β = −1.05; 95% CI: −1.62 to −0.48). No significant associations were observed between the domestic food environment and food insecurity, nor between the community food environment and the remaining model variables. No significant indirect associations were identified, indicating no mediation through food environment perceptions. Greater food insecurity severity was positively associated with maternal BMI (β = 0.19 95% CI: 0.002–0.37), and maternal BMI was positively associated with child BMI-for-age (β = 0.25 95% CI: 0.09–0.39). **Conclusions**: Social and housing conditions play a central role in shaping food security and nutritional outcomes, while food environment perception showed limited relevance. These findings reinforce the need for intersectoral public policies addressing social inequalities and promoting healthy eating.

## 1. Introduction

The human right to adequate food (HRAF) ensures that individuals have access to sufficient, nutritious, and culturally appropriate food. Food security (FS) constitutes a practical instrument for the realization of this right, incorporating, beyond access, aspects related to food quality and stability [1,2].

Among the various factors contributing to food insecurity (FI), the globalization of the food system stands out, as it has promoted increased consumption of ultra-processed foods (UPF) and reduced intake of fresh and minimally processed foods [3]. The local food environment, in which consumers decide which foods to purchase, represents a strategic point for interventions aimed at reorienting the food system toward more sustainable diets [4,5].

Child malnutrition is influenced by multiple factors, including low parental education, inadequate sanitation and hygiene practices, and large household size. In this context, stunting constitutes a critical metric of chronic undernutrition, and is equally impacted by environmental, socioeconomic, and cultural conditions [6]. The Food and Agriculture Organization of the United Nations (FAO) identified a worsening situation in Brazil between 2012 and 2024 in the categories of inadequate growth and overweight among children under five years of age. Among adults, a similar deterioration in obesity rates was observed between 2012 and 2022 [7], reinforcing the relevance of studies that investigate the factors driving this problem.

Another important concept is that of perception, as it involves the interaction of human senses, as well as the recognition, organization, and interpretation of information, and influences the way people understand and interact with their environment [8]. Perception is subject to subjective interpretations, varying according to individual personal experience and social context. In this way, parameters such as food access, availability, diversity, nutritional adequacy, and cost directly affect this perception [9].

Despite the growing recognition of food environments as determinants of dietary practices [10,11], evidence remains limited regarding how social and housing conditions interact with food environment perceptions and food security, particularly in contexts of social vulnerability in Northeastern Brazil. Furthermore, studies that simultaneously explore these relationships through structural modeling remain scarce.

The theoretical pathways between the variables examined in this study have been supported by well-developed frameworks. According to the Social Determinants of Health framework [12], socioeconomic and housing factors are considered structural determinants that create living conditions and affect health outcomes throughout life. Extending from this framework, the FAO [13] food security framework defines food security as consisting of four interlinked dimensions, including availability, access, utilization, and stability, all of which are affected by the aforementioned structural factors, such as income, education, and housing. Moreover, the United Nations International Children’s Emergency Fund (UNICEF) [14] framework on malnutrition has determined the food security dimension as one of the proximal determinants of children’s nutritional status along with care and health environments. Lastly, food environment frameworks [4] explain how the food environment (household and community) serves as the intermediary mechanism in the relationship between structural factors and diet. Together, these frameworks support the investigation of direct and indirect associations among social and housing conditions, food environments, food insecurity, and nutritional outcomes.

Given this context, the present study aimed to analyze the direct and indirect associations between social and housing determinants, food environment perception, food security, and nutritional outcomes of children and mothers, considering the mediating role of caregivers’ perceptions of the community and domestic food environment.

Accordingly, the following sections describe the study methods, present the findings from the generalized structural equation models, discuss the results in light of the existing literature, and summarize the main conclusions and public health implications.

## 2. Materials and Methods

### 2.1. Study Design

A cross-sectional study conducted in the municipality of Vitória de Santo Antão, Pernambuco, between October 2022 and March 2023. The municipality has approximately 134,084 inhabitants and a population density of around 398.38 inhabitants per square kilometer [15], as well as a per capita GDP of approximately US$6032.00 [15]. In recent years, the municipality has developed in various respects; however, this growth, when unplanned, can intensify vulnerability situations in certain population groups.

The sample consisted of children aged 0 to 9 years followed by Family Health Units (FHU) and/or beneficiaries of cash transfer programs (CTP). Children with neurological or motor disorders that prevented physical assessment, as well as those older than nine years, were excluded from the study.

### 2.2. Sample Size and Sampling Procedures

For sample sizing, the prevalence of excess weight in children aged 0 to 9 years in the municipality was used as a parameter: 27.7% among participants in CTP and 10.9% among non-participants [16]. The design considered a significance level of 95% and study power (1–β) of 80%. Additionally, a 20% margin of error was added for possible losses, resulting in a final sample of 314 children.

During the data collection period, the municipality had 36 Basic Health Units (BHU), distributed with approximately 70% in the urban area and 30% in the rural area. Based on this proportion, the study was conducted in ten units—seven located in the urban area and three in the rural area—selected by random draw, with approximately 30 interviews conducted at each FHU. Calculations were performed using Epi Info™ StatCalc, version 6.04 (Centers for Disease Control and Prevention, Atlanta, GA, USA) software for Windows.

### 2.3. Data Collection Instrument

Data were collected through a structured questionnaire administered to the children’s caregivers, including socioeconomic information, anthropometric assessment of children and mothers (weight and height), measurement of food insecurity using the Brazilian Food Insecurity Scale (BFIS), and assessment of food environment perception. The Domestic Food Environment (DFE) encompassed the availability of Fresh and Minimally Processed Foods (FMPF) and UPF in the home, while the Community Food Environment (CFE) covered aspects related to the availability, quality, variety, and accessibility of FMPF and UPF in the residential neighborhood [17,18].

### 2.4. Anthropometric Outcomes

Weight and height (stature or length) were measured in duplicate, following the techniques recommended by Lohman et al. [19]. These results formed the basis for calculating the nutritional status of the children, assessed through the Height-for-Age (H/A) and Body Mass Index-for-Age (BMI/A) indicators, adjusted for sex. Anthropometric indicators were expressed as Z-scores and calculated using Anthro software (version 3.2.2) for children under 5 years and Anthro Plus (version 1.0.3) for children aged 5 to 9 years.

For the H/A indicator, the following cutoff points were adopted: stunting (H/A < −2 Z-score) and adequate height (H/A ≥ −2 Z-score) [20]. Nutritional classification was carried out according to the criteria of the Food and Nutrition Surveillance System [21], considering BMI/A: underweight (BMI/A < −2 Z-score); eutrophic (−2 SD ≤ BMI/A < +1 SD); overweight risk (+1 SD ≤ BMI/A < +2 SD); overweight (+2 SD ≤ BMI/A < +3 SD); and obesity (BMI/A ≥ +3 SD).

Maternal nutritional status was assessed using BMI, calculated as the ratio of weight (kg) to the square of height (m^2^), classified according to World Health Organization (WHO) criteria: underweight (BMI < 18.5); eutrophic (BMI between 18.5–24.9); overweight (BMI between 25–29.9); and obesity (BMI ≥ 30) [22].

### 2.5. Endogenous and Exogenous Variables

#### 2.5.1. Social and Housing Conditions (SHC)

The socioeconomic situation was assessed based on a poverty measurement instrument that investigated indicators such as: education (years of schooling); income (wage brackets); occupational status (formal employment, informal/self-employment, no paid work); social class [23]; and housing conditions, including type of dwelling, number of rooms and bedrooms, and floor type (cement, ceramic, or wood).

Although there is no direct scientific evidence linking housing characteristics—such as floor type, number of rooms, and number of bedrooms—to food consumption, these variables function as proxies for the socioeconomic and territorial conditions of households. In contexts of social vulnerability, housing conditions reflect material deprivations and structural inequalities that indirectly influence eating behavior and nutritional status by affecting access to resources, household stability, and conditions for acquiring, storing, and preparing food [24].

The operationalization of socioeconomic and housing variables as a single latent construct reflects a theoretical choice grounded in the social determination of health framework, as developed in Latin American critical epidemiology [25]. Within this perspective, housing conditions and socioeconomic position are not viewed as independent dimensions but rather as interconnected expressions of the same process of social reproduction, encompassing the material, political, and cultural conditions that shape the living standards and well-being of social groups. Treating these dimensions separately would artificially fragment a structural reality that is experienced as an integrated whole. Accordingly, the Social and Housing Conditions (SHC) latent construct was specified to capture this underlying unity, consistent with the theoretical understanding that socioeconomic and housing inequalities share common structural determinants. This operationalization has also been adopted in previous studies based on the same theoretical framework [26,27,28,29,30].

#### 2.5.2. Perception of the Domestic Food Environment (DFE) and Community Food Environment (CFE)

Food environment perception was assessed using an instrument from the Brazilian National Survey on Child Feeding and Nutrition (ENANI) [17,18]. For the DFE, nine questions were applied regarding food availability in the home over the previous 30 days—four related to FMPF and five UPF. Responses were recorded on an ordinal frequency scale (never, rarely, sometimes, almost always, always).

For the CFE, seven questions were applied related to neighborhood perception—defined as the area accessible within 20 min on foot or 5 to 10 min by vehicle—covering availability, quality, variety, and price of FMPF (four items), as well as accessibility, variety, and price of UPF (three items), with responses on an agreement scale (strongly disagree to strongly agree).

Scores were constructed based on the methodology proposed by Castro Júnior [31]. For FMPF, responses received increasing scores (1 to 5), so that higher values indicated greater availability and better perceived quality. For UPF, the scoring was reversed, so that higher values represented lower availability or accessibility of these foods.

In the DFE, the FMPF score (S-FMPF) ranged from 4 to 20 points and the UPF score (S-UPF) from 5 to 25 points. In the CFE, the S-FMPF ranged from 4 to 20 points and the S-UPF from 3 to 15 points. To ensure comparability across scores, all were standardized on a 0 to 10 scale by dividing total values by their respective adjustment factors.

From these scores, two composite indicators were constructed: the Domestic Food Environment Healthfulness Perception Index (DFEHPI) and the Community Food Environment Healthfulness Perception Index (CFEHPI), calculated as the simple average of the standardized FMPF and UPF scores. In both cases, higher values indicated healthier food environments, characterized by greater availability of FMPF (fruits, vegetables, greens, and beans/other legumes) and lower presence of UPF (industrialized juice, soft drinks, cookies, packaged snacks, and sweets).

#### 2.5.3. Food Insecurity

Food security and insecurity status was assessed using the BFIS, comprising 14 questions. Each affirmative response corresponds to one point, and the total sum represents the scale score. In households with individuals under 18 years, the classification was as follows: 0 = food security; 1–5 = mild food insecurity; 6–9 = moderate food insecurity; and 10–14 = severe food insecurity [32,33].

### 2.6. Statistical Analysis

The analysis of Direct Effects (DE) and Indirect Effects (IE) of social and housing determinants on food security and the nutritional status of children and mothers was conducted using Generalized Structural Equation Modeling (GSEM). This approach was adopted due to the complex nature of the phenomenon under investigation and the need to integrate, in a single analytical model, latent and observable variables with different statistical natures. This approach was considered particularly appropriate for investigating simultaneous pathways between structural determinants, mediating variables, and nutritional outcomes.

Social and housing determinants were operationalized as a latent construct, referred to as Social and Housing Conditions (SHC), composed of observable indicators related to education, income, social class, occupational status, and housing conditions—including floor type, number of rooms, and number of bedrooms. These indicators were treated as complementary dimensions of a single structural construct, representing different expressions of socioeconomic vulnerability at the household level [34,35]. Measurement models were initially fitted to assess the adequacy of these indicators in composing the latent construct.

In the structural model, direct and indirect relationships were tested between social and housing determinants, perceptions of the community food environment (CFE) and domestic food environment (DFE), food security, and the anthropometric indicators height-for-age (H/A), BMI-for-age (BMI/A), and maternal BMI (BMI/mother). CFE and DFE perceptions were included as mediating variables, allowing exploration of the mechanisms by which structural factors are associated with nutritional outcomes along the proposed theoretical framework.

The study models were guided by the following hypotheses: (i) social and housing determinants, derived from factor analysis, are associated with perceptions of the community and domestic food environments, compromising food security and being associated with height deficit in children; and (ii) social and housing determinants affect perceptions of the community and domestic food environments, impacting food security and being associated with poor nutrition in the mother-child dyad. Both models share the same theoretical structure of relationships between variables, differing only in their final outcome—the first related to height-for-age and the second to maternal and child BMI indicators.

The conceptual organization and directionality of relationships between exogenous and endogenous variables were represented through a Directed Acyclic Graph (DAG), which guided the formulation of the structural model (Supplementary Figure S1). Although GSEM allows the representation of relationships compatible with causal hypotheses, the primary goal of the analysis was to assess patterns of association between observed and latent variables, consistent with the cross-sectional study design.

Descriptive analyses were performed in IBM SPSS Statistics, version 26.0 (IBM Corp., Armonk, NY, USA), and GSEM models were estimated in R software version 4.3.3 (R Core Team, R Foundation for Statistical Computing, Vienna, Austria), using appropriate routines for generalized structural equation modeling compatible with variables of different distributions. A significance level of *p* < 0.05 was adopted. Results were presented using unstandardized coefficients along with their respective 95% confidence intervals (95% CI), allowing interpretation of the direction and magnitude of the associations estimated in the model.

The adequacy of the factor analysis and the latent construct structure was verified using the Kaiser-Meyer-Olkin (KMO) measure—with values above 0.5 considered adequate—and Bartlett’s test of sphericity, with *p* < 0.05 taken as indicative of the correlation matrix’s suitability for factor analysis [36]. Model fit was evaluated using plausibility and parsimony indicators, including Root Mean Square Error of Approximation (RMSEA) and Standardized Root Mean Square Residual (SRMSR), with values ≤ 0.08 considered acceptable [37,38]. These indices were used as complementary fit references, considering the limitations of their application in models with non-continuous outcomes.

For a more detailed understanding of the estimated relationships, unstandardized direct and indirect effects were calculated with their respective 95% confidence intervals, allowing evaluation of the intermediary pathways between constructs [39,40]. The unstandardized coefficient expresses the effect of the exogenous variable on the endogenous variable, assuming all other model variables remain constant [39,41].

### 2.7. Ethical Considerations

This project was approved by the Research Ethics Committee of the Health Sciences Center at the Federal University of Pernambuco (UFPE), under Ethics Submission Certificate (CAAE) No. 74287223.6.0000.5208.

## 3. Results

The data presented in Table 1 describe the main socioeconomic, housing, and nutritional characteristics of the sample. Most caregivers had completed only primary education (67.8%) and had no paid employment (62.7%). Approximately half of the families belonged to the lowest socioeconomic strata (48.1%) and reported a monthly household income between half and one minimum wage (48.4%). Regarding housing conditions, 53.2% of families lived in rented or borrowed dwellings, 49.7% of households had unfinished flooring, 52.5% had up to five rooms, and 79.2% had up to two bedrooms.

Concerning the nutritional profile, 44.2% of households experienced moderate to severe food insecurity, 63.9% of mothers were classified as overweight, and 36.9% of children showed impairment in the height-for-age index. Taken together, these findings highlight a context of high social, housing, and nutritional vulnerability.

The estimated coefficients indicated a negative association between the Social and Housing Conditions (SHC) factor and food insecurity severity. The food insecurity variable was coded on an increasing ordinal scale, with higher values representing greater severity. Therefore, the negative coefficient indicates that more favorable social and housing conditions were associated with lower food insecurity severity.

**Figure 1:**
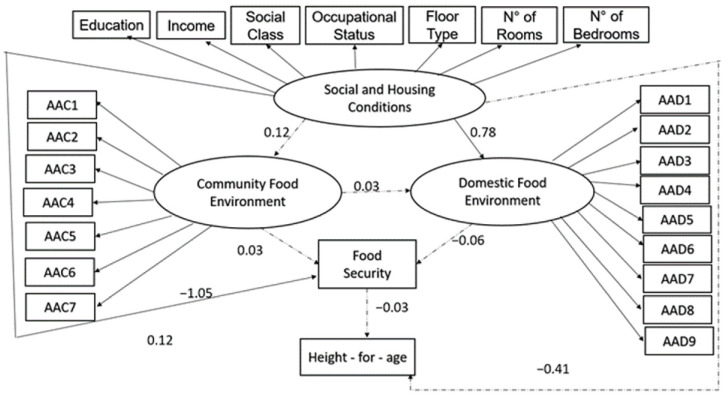
Complex structural model fitted by GSEM, representing the social and housing determinants associated with domestic and community food environments, food security, and the height-for-age index in children. Vitória de Santo Antão, Pernambuco, Brazil, 2022–2023. Values presented correspond to unstandardized coefficients. Dashed arrows indicate statistically non-significant relationships (*p* > 0.05).

**Figure 2:**
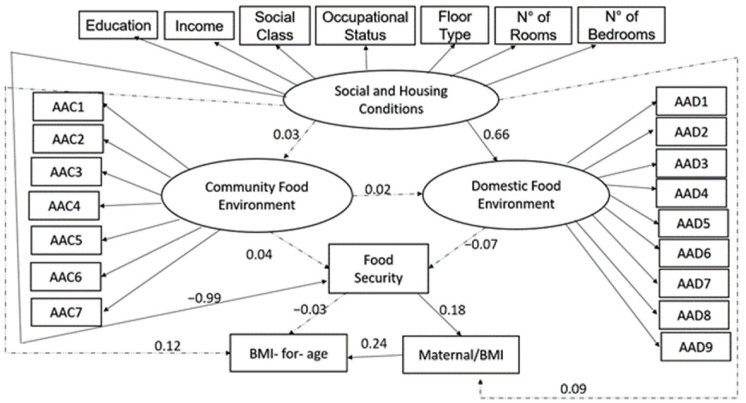
Complex structural model fitted by GSEM representing the social and housing determinants associated with domestic and community food environments, food security, and the indicators of maternal BMI and child BMI-for-age. Vitória de Santo Antão, Pernambuco, Brazil, 2022/2023. Values presented correspond to unstandardized coefficients Dashed arrows indicate statistically non-significant relationships (*p* > 0.05).

The assessment of the latent construct SHC yielded a KMO value of 0.705 and Bartlett’s test of sphericity with *p* < 0.001, indicating adequacy of the factor structure for analysis [36]. The RMSEA and SRMR indices were 0.057 and 0.054 in Model 1 (Figure 1), and 0.051 and 0.056 in Model 2 (Figure 2), respectively, suggesting acceptable model fit [37,38].

The structural models indicated that SHC was consistently associated with the domestic food environment (DFE) and with food security. Considering the coding of the food security variable, the negative coefficients observed indicate that better structural conditions are associated with lower levels of food insecurity.

A negative and significant association was also observed between SHC and FS (Model 1: DE = −1.05; 95% CI: −1.62 to −0.48; *p* < 0.001; Model 2: DE = −0.99; 95% CI: −1.52 to −0.46; *p* < 0.001), further confirming that better structural conditions reduce the likelihood of food insecurity.

No statistically significant associations were observed between DFE and FS, nor between the community food environment (CFE) and other model variables. Additionally, no significant indirect effects (IE) were identified, indicating the absence of mediation.

In the model including anthropometric outcomes, a significant association was found between food security and maternal BMI (DE = 0.19 95% CI: 0.002–0.37; *p* = 0.047), as well as between maternal BMI and child BMI-for-age (DE = 0.25; 95% CI: 0.09–0.39; *p* = 0.002). Given the coding of the FS variable, this result suggests that higher levels of food insecurity are associated with worse maternal nutritional outcomes, with subsequent repercussions on child nutritional status.

Table 2 and Table 3 present the unstandardized direct and indirect coefficients, *p*-values, and respective 95% confidence intervals for Models 1 and 2.

## 4. Discussion

The current investigation sought to use generalized structural equation modeling to analyze the relationship between SHC, perceptions of the domestic and community food environment, food security, and nutrition outcomes for children and mothers from one Brazilian municipality located in the northeastern part of the country. The findings indicate that social and housing conditions are connected with food security and perception of the domestic food environment but not mediated by perceptions of either community or domestic food environment. Overall, structural conditions showed stronger associations with food insecurity than the proposed perceptual mediating pathways.

The observed association between SHC, the domestic food environment (DFE), and food security reinforces the role of structural conditions in shaping dietary practices and regular access to adequate food. Factors such as income, education level, employment status, and housing conditions influence the household’s food organization, affecting the availability of fresh and minimally processed foods while increasing the presence of ultra-processed foods (UPF), particularly in contexts of social vulnerability [42,43,44].

In this regard, the direct relationship between better social and housing conditions and lower food insecurity observed in this study corroborates evidence that socioeconomic inequalities, labor instability, precarious housing, and the absence of social protection are central determinants of food insecurity [45,46,47,48,49,50,51,52]. This direct relationship likely reflects a shared structural pathway rather than a sequential one: limited household income constrains both housing quality and food purchasing power simultaneously, forcing trade-offs between housing-related expenses and food expenditures [53,54]. In the Brazilian context, income remains the most consistent determinant of household food insecurity even after adjusting for other socioeconomic factors [55], supporting the interpretation that SHC and food security are jointly shaped by underlying poverty conditions. Although the DFE was associated with SHC, its perception showed no significant relationship with food security in the analyzed model, suggesting that perceptive measures of the home environment may not fully capture the deeper material constraints that determine adequate food access in socially vulnerable populations.

From a theoretical standpoint, food environments constitute a pathway connecting structural conditions and health outcomes by influencing dietary behavior. In this context, unequal social structures may promote the formation of obesogenic food environments [56,57]. However, in the present study, the community food environment showed no significant associations with other model variables, nor with food security. These results may reflect inherent limitations of relying exclusively on perceptive measures of the food environment, which do not necessarily capture actual food acquisition practices, habitual shopping locations, daily mobility, or objective territorial characteristics such as the geographic availability of food outlets, walkability, and physical accessibility to food [58]. Therefore, future studies may benefit from integrating perceptive measures, observational assessments of the food environment, and food purchasing behavior indicators, enabling a more precise characterization of the interaction between territorial context, food access, and dietary intake [59,60].

Similarly, no association was observed between the community food environment and food security. Although the literature indicates that limited availability of healthy foods and a greater concentration of UPF-dominant establishments in low-income areas are associated with food and nutritional insecurity [61,62,63,64,65], the findings of this study suggest that, in the analyzed context, these factors may not have been adequately captured by the perceptive measures employed. The systematic review conducted by Westbury et al. [66] further confirms that, in low- and middle-income countries, the structure of the urban food environment is associated with inadequate dietary patterns, although this relationship may vary according to context and measurement approach.

No statistically significant indirect associations were identified, and therefore the hypothesized mediating pathways were not supported in this sample. This finding suggests that the relationship between social and housing conditions and the analyzed outcomes occurs predominantly through direct pathways, rather than through the intermediate mechanisms represented by perceptions of food environments. This result may also reflect a relative homogeneity of food environment exposure among highly vulnerable populations, reducing the variability needed to discriminate associations between environmental perception and food security.

In the model that incorporated maternal and child BMI indicators, significant associations were observed between food security and maternal BMI, as well as between maternal BMI and child BMI-for-age. These findings highlight that the nutritional outcomes of the mother-child dyad share social, dietary, and household determinants. The scoping review conducted by O’Meara et al. [67] indicates that structural, ecosocial, and individual factors influence women’s dietary practices. Complementarily, a study with Iranian mothers identified food security as one of the primary determinants of maternal BMI [68].

The nutritional profile of the mother-child dyad also reveals the coexistence of food insecurity and excess weight, reflecting the multiple forms of malnutrition characteristic of contexts undergoing nutritional transition. This phenomenon has been associated with increased consumption of ultra-processed foods, driven by factors such as cost, convenience, and availability, reinforcing the need for policies that simultaneously address undernutrition and excess weight [69,70]. The coexistence of food insecurity and overweight observed in this study aligns with the contemporary overlap of multiple forms of malnutrition, in which food deprivation coexists with dietary patterns based on ultra-processed products. This phenomenon demonstrates that food insecurity is not limited to quantitative food insufficiency, but may occur simultaneously with the consumption of nutritionally poor-quality diets [10,63].

In the context of the Brazilian nutritional transition [71,72], the present study reinforces the need to advance toward more contextualized analyses of food and diet, considering not only food availability but also the degree and purpose of food processing. Recent evidence indicates that ultra-processed foods have progressively replaced dietary patterns historically based on fresh or minimally processed foods and traditional culinary preparations, a phenomenon associated with deteriorating diet quality and increasing global burden of diet-related diseases [73]. In this scenario, the expansion of UPF undermines the sustainability of food systems, intensifies inequalities in access to adequate and healthy food, and disproportionately affects socially vulnerable groups [7,73,74].

This study has limitations that must be considered. The cross-sectional design precludes establishing temporality between exposures and outcomes, thereby limiting causal interpretations. Furthermore, the food environment measures were based on the perceptions of caregivers and may not fully reflect objective territorial characteristics or actual food acquisition behaviors. Nevertheless, the use of a standardized instrument and prior training of interviewers aimed to reduce potential information biases. Additionally, data collection in a post-pandemic context may reflect social and dietary conditions still influenced by the prolonged effects of the COVID-19 pandemic.

By integrating social, housing, dietary, and nutritional dimensions within a single structural model, this study broadens traditional approaches centered exclusively on income or food availability, highlighting the multidimensional nature of food insecurity. However, given the cross-sectional design, these findings should be interpreted as associations compatible with the proposed theoretical model, rather than as causal relationships.

Among the strengths of this study, the use of GSEM stands out, as it allowed for the simultaneous analysis of relationships between observed and latent variables, along with the incorporation of social, housing, dietary, and nutritional dimensions within the same analytical model. The study also contributes by examining families in social vulnerability enrolled in social protection programs, providing valuable insights for the debate on social protection, food environments, and maternal and child nutrition.

Future studies should integrate objective measures of the food environment, such as geographic mapping of food outlets and walkability indices, alongside perceptual measures, to more comprehensively characterize the relationship between territorial context and food access. Longitudinal studies are also needed to establish temporality between structural living conditions, food environments, food insecurity, and nutritional outcomes. Additionally, qualitative approaches may help elucidate the mechanisms through which highly vulnerable populations navigate food insecurity in contexts of limited food environment variability.

## 5. Conclusions

The findings of this study indicate that more favorable social and housing conditions were associated with lower food insecurity severity and a healthier perceived household food environment among mothers and children living in a municipality in Northeast Brazil. In contrast, perceived food environments did not emerge as relevant mediating mechanisms in the proposed model, suggesting that structural living conditions played a more prominent role in explaining food insecurity than the perceptual pathways initially hypothesized.

Although these findings derive from a cross-sectional study and should therefore be interpreted as associations consistent with the proposed theoretical model rather than as evidence of causal relationships, they reinforce the understanding of food insecurity as a multidimensional phenomenon influenced by material living conditions and broader social inequalities [49,50,73]. In this context, housing conditions should be interpreted as one component of a broader set of structural factors reflecting socioeconomic disadvantage rather than as isolated determinants of food insecurity [50,52].

These findings add to the growing body of evidence on food environments and food security in low- and middle-income countries, indicating that interventions focused exclusively on improving food environments may be insufficient if they are not accompanied.

## Figures and Tables

**Table 1:** Characterization of socioeconomic, demographic, and nutritional factors among families of children who are beneficiaries and non-beneficiaries of the Bolsa Família Program. Vitória de Santo Antão, Pernambuco, Brazil, 2022/2023.

	*N* = 314	%	95% CI
Caregiver’s education level			
Never attended school	8	2.5	0.77–4.22
Up to 8 years of schooling	93	29.6	24.5–34.6
>9 years of schooling	213	67.8	62.6–72.9
Employment status			
No paid employment	197	62.7	57.3–68.0
Informal/self-employed work	68	21.7	17.1–26.2
Formal employment	49	15.6	11.5–19.6
Socioeconomic classification (ABEP)			
Up to B2	18	5.7	3.13–8.26
C1	45	14.4	10.5–18.2
C2	100	31.8	26.6–36.9
D–E	151	48.1	42.5–53.6
Household income (MW)			
Up to half MW	98	31.2	26.0–36.3
Half to 1 MW	152	48.4	42.8–53.9
>1 MW	64	20.4	15.9–24.8
Housing tenure			
Owned	147	46.8	41.2–52.3
Rent-free/ceded	49	15.6	11.5–19.6
Rented	118	37.6	32.2–42.9
Floor Type			
Ceramic/concrete slab	158	50.3	44.7–55.8
Cement	146	46.5	40.9–52.0
Other	10	3.2	1.25–5.14
Number of rooms			
Up to 5	165	52.5	46.9–58.0
≥6	149	47.5	41.9–53.0
Number of bedrooms			
Up to 2	249	79.3	74.7–83.6
≥3	65	20.7	16.2–25.1
Food security and insecurity (FI)			
Food security	39	12.4	8.7–16.0
Mild FI	136	43.3	37.8–48.7
Moderate FI	100	31.8	26.6–36.9
Severe FI	39	12.4	8.7–16.0
Maternal BMI * (*N* = 272)			
Underweight	8	2.9	0.90–4.8
Normal weight	90	33.1	27.5–38.6
Overweight	79	29.0	23.6–34.3
Obesity	95	34.9	29.2–40.5
Height-for-age			
Stunted	116	36.9	31.5–42.2
Adequate	198	63.1	57.7–68.4
BMI-for-age			
Underweight	62	19.7	15.3–24.0
Normal weight	102	32.5	27.3–37.6
Overweight risk	69	22.0	17.3–26.4
Overweight	29	9.2	6.0–12.3
Obesity	52	16.6	12.4–20.7

MW: Minimum wage; FI: Food insecurity; BMI: Body mass index. * Missing data: Maternal BMI, *N* = 42. Percentages were calculated based on valid observations for each variable.

**Table 2:** Unstandardized direct and indirect coefficients and significance values for the structural paths of Model 1.

Relationship	Type	Effect	*p*-Value	95% CI Lower	95% CI Upper
SHC–CFE	Direct	0.129	0.633	−0.40	0.66
SHC–DFE	Direct	0.789	0.005	0.23	1.34
SHC–FS	Direct	−1.053	0.000	−1.62	−0.48
SHC–H/A	Direct	−0.413	0.082	−0.879	0.053
CFE–DFE	Direct	0.034	0.524	−0.71	0.14
DFE–FS	Direct	−0.063	0.149	−0.14	0.02
CFE–FS	Direct	0.035	0.382	−0.04	0.11
FS–H/A	Direct	−0.032	0.629	−0.16	0.09
SHC–DFE–FS	Indirect	−0.050	0.162	−0.12	0.02
SHC–DFE–FS–H/A	Indirect	0.002	0.643	−0.00	0.00
SHC–CFE–FS	Indirect	0.005	0.698	−0.02	0.03
CFE–FS–H/A	Indirect	−0.001	0.688	−0.00	0.00
DFE–FS–H/A	Indirect	0.002	0.641	−0.00	0.00

SHC: Social and housing conditions; DFE: Domestic food environment; CFE: Community food environment; FS: Food security; H/A: Height-for-age.

**Table 3:** Unstandardized direct and indirect coefficients and significance values for the structural paths of Model 2.

Relationship	Type	Effect	*p*-Value	95% CI Lower	95% CI Upper
SHC–CFE	Direct	0.034	0.899	−0.49	0.56
SHC–DFE	Direct	0.667	0.013	0.14	1.19
SHC–FS	Direct	−0.994	<0.001	−1.52	−0.46
SHC–Maternal BMI	Direct	0.091	0.763	−0.50	0.68
SHC–Child BMI	Direct	0.129	0.738	−0.62	0.88
CFE–DFE	Direct	0.020	0.727	−0.09	0.13
DFE–FS	Direct	−0.079	0.093	−0.17	0.01
CFE–FS	Direct	0.046	0.296	−0.04	0.13
FS–Maternal BMI	Direct	0.187	0.047	0.00	0.37
FS–Child BMI	Direct	−0.039	0.749	−0.27	0.19
Maternal BMI–Child BMI	Direct	0.245	0.002	0.09	0.39
SHC–CFE–FS	Indirect	0.002	0.902	−0.02	0.02
SHC–DFE–FS	Indirect	−0.053	0.122	−0.11	0.01
SHC–FS–Child BMI	Indirect	0.039	0.749	−0.19	0.27
SHC–FS–Maternal BMI–Child BMI	Indirect	−0.046	0.126	−0.10	0.01
SHC–FS–Maternal BMI	Indirect	−0.186	0.080	−0.39	0.02

SHC: Social and housing conditions; DFE: Domestic food environment; CFE: Community food environment; FS: Food security; BMI: Body mass index.

## Data Availability

The data presented in this study are not publicly available due to ethical restrictions but are retained for a period of five years in accordance with institutional guidelines. Data may be made available upon reasonable request to the corresponding author.

## Supplementary Materials

The following supporting information can be downloaded at: https://www.mdpi.com/article/10.3390/ijerph23070936/s1, Figure S1: Proposed conceptual model for analysis using generalized structural equation modeling (GSEM), illustrating the hypothetical relationships between latent and manifest variables before model estimation.

## Abbreviations

The following abbreviations are used in this manuscript:

HRAF	Human Right to Adequate Food
FS	Food Security
FI	Food Insecurity
UPF	Ultra-Processed Foods
FAO	The Food and Agriculture Organization of the United Nations
UNICEF	United Nations International Children’s Emergency Fund
FHU	Family Health Units
CTP	Cash Transfer Programs
BHU	Basic Health Units
BFIS	Brazilian Food Insecurity Scale
FMPF	Fresh and Minimally Processed Foods
CFE	Community Food Environment
H/A	Height-for-Age
BMIA	Body Mass Index-for-Age
FNSS	Food and Nutrition Surveillance System
WHO	World Health Organization
SHC	Social and Housing Conditions
DFEHPI	Domestic Food Environment Healthfulness Perception Index
CFEHPI	Community Food Environment Healthfulness Perception Index
DE	Direct Effects
IE	Indirect Effects
GSEM	Generalized Structural Equation Modeling
DAG	Directed Acyclic Graph
KMO	Kaiser-Meyer-Olkin
RMSEA	Root Mean Square Error of Approximation
SRMSR	Standardized Root Mean Square Residual
UFPE	University Federal of Pernambuco
CAAE	Ethics Submission Certificate
DFE	Domestic Food Environment
