# Supplementary material for: Food Environments and Food Security Among Mothers and Children in Northeast Brazil: The Role of Social and Housing Conditions
Source: Int J Environ Res Public Health. 2026 Jul 22;23(7):936. doi: 10.3390/ijerph23070936 (PMC13409937; doi:10.3390/ijerph23070936)

Supplementary File

Manuscript Title: Social and housing determinants, food environments and food security among mothers and children in Northeast Brazil: a generalized structural equation modeling approach

All figures are original works created by the authors and have not been previously published.

Figure S1. Proposed conceptual model for analysis using generalized structural equation modeling (GSEM), illustrating the hypothetical relationships between latent and manifest variables before model estimation.

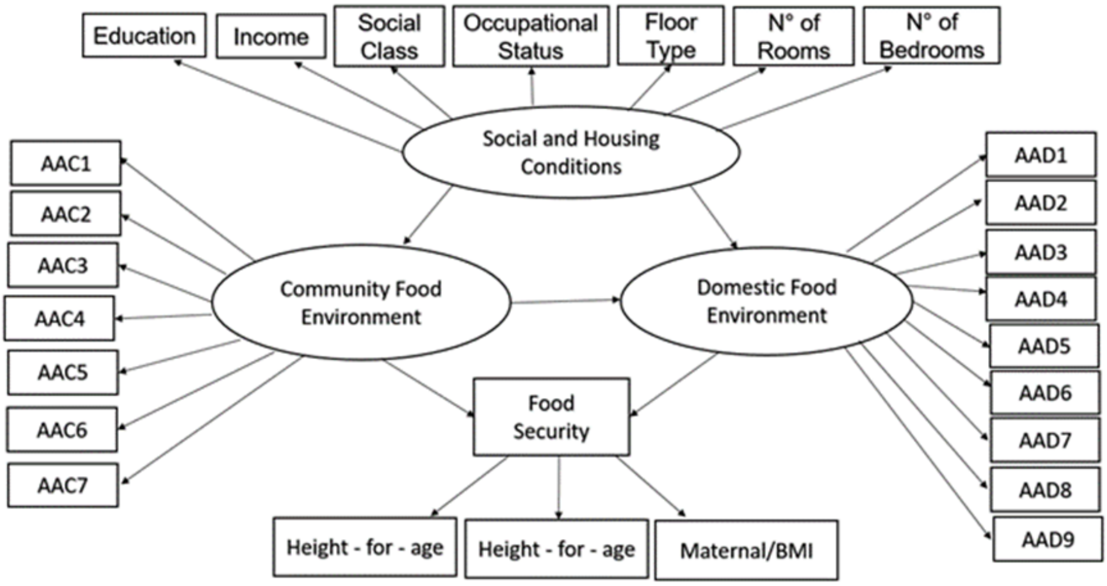

Supplement: Supplementary file 1 [file ijerph-23-00936-s001.zip › ijerph-4396798-supplementary.pdf]
